# The Story of the Insane from Year to Year

**Published:** 1902-04-12

**Authors:** 


### THE STORY OF THE INSANE FROM YEAR TO YEAR.

Fourteenth Annual Series.

### LEBANON HOSPITAL FOR THE INSANE AT ASFURIYEH NEAR BEYROUT.

This claims to be the first asylum for the insane in Bible lands, and it now issues its third annual report; but this report states that the asylum was opened for the reception of patients on August 6th, 1900. Up to March 31st, 1901, 54 patients were admitted, 38 of these being men, and 16 women. Of the admissions 9 have been discharged recovered and 4 relieved, a not unsatisfactory result considering that the large majority of the cases admitted were in a chronic stage of the disease. There were 2 deaths. The patients came from districts widely separated, but the most were from Lebanon and Beyrout. Aleppo sent 1, Damascus 1, Egypt 3, and Cyprus 1, Their religions, also, show a wide range. There were 4 Protestants, 15 of the Greek Church, 9 Catholics, 20 Maronites, and 4 Moslems. Jews and Druses were also represented. The treatment of the insane in Syria is in a very primitive state, and this asylum cannot fail to do much good, not only to those who are treated within its walls, but by force of example to those who are still kept in chains by their relations or by the authorities. Although some of the patients may be able to pay a little towards their maintenance, the majority cannot, and the hospital must rely almost solely on contributions from the charitable. Each patient costs about £25 a year, which is a very moderate sum, as establishment charges of various kinds have to be distributed over a small number only. The male wards are now full, and a small block for acute cases is urgently needed. It is to be hoped that sufficient funds will soon be forthcoming to enable the trustees to erect this block and so extend the usefulness of an institution the necessity for which there can be no doubt. The hospital is entirely non-sectarian. The Treasurer is Sir Richard Tangye, 35 Queen Victoria Street, London, E.C.

### DERBY BOROUGH ASYLUM.

There were 300 patients in residence on January 1st, 1900, and 306 on December 31st. During the year 94 patients were admitted, 34 were discharged recovered, 8 relieved, and 44 died. The average number resident was 304, and the total number of persons under treatment was 394. Calculated on the admissions the recoveries stand at 38·2 per cent., and calculated on the average number resident the deaths stand at 14·4 per cent. The recoveries are under the average at Derby for the last 12 years, and the deaths are above the average, the former being 44·7 and the latter 11·2. Of the year's deaths 25 were caused by cerebral and spinal diseases, 3 by consumption, and 1 by pneumonia. Various moral causes such as domestic trouble and adverse circumstances account for the insanity in 23 of the cases admitted, intemperance in drink for 21, and hereditary influence for 31. The greatest number of patients admitted in any one month was in May when 14 new cases appeared, and the lowest was in October when there were only 4. A table is given which shows the nationality of the patients. Of the total 254 were English, 5 Scotch, 30 Irish, 15 Welsh, 1 was American, and 1 Spanish. The high mortality was chiefly owing to the number of general paralytics and not to any epidemic disease, for although influenza broke out and attacked 117 people it was only fatal in 1 case. The women's wards are nearly full, and it will soon be necessary either to discharge the private patients or to build further accommodation. Dr. Macphail is in favour of adding a separate building for private patients, and there cannot be a doubt that this is the proper course to take, for it would be alike beneficial to the patients, the asylum, and the ratepayers. The committee express "their entire satisfaction with the manner in which Dr. Macphail continues to discharge the responsible and onerous duties which devolve upon him." The weekly charge for maintenance is 10s. 6d. per head. Out-borough cases pay from 14s. to 17s. 6d., and private patients the same.

